# Systemic factors in young human serum influence *in vitro* responses of human skin and bone marrow-derived blood cells in a microphysiological co-culture system

**DOI:** 10.18632/aging.206288

**Published:** 2025-07-25

**Authors:** Johanna Ritter, Cassandra Falckenhayn, Minyue Qi, Leonie Gather, Daniel Gutjahr, Johannes Schmidt, Stefan Simm, Stefan Kalkhof, Janosch Hildebrand, Thomas Bosch, Marc Winnefeld, Elke Grönniger, Annette Siracusa

**Affiliations:** 1 Beiersdorf AG, Research and Development, Hamburg, Germany; 2 Institute for Bioanalytics, Coburg University of Applied Sciences and Arts, Coburg, Germany; 3 Proteomics Unit, Department Preclinical Development and Validation, Fraunhofer Institute for Cell Therapy and Immunology, Leipzig, Germany; 4 Zoological Institute, Kiel University, Kiel, Germany

**Keywords:** skin rejuvenation, microphysiological systems, systemic factors, bone marrow model, human serum

## Abstract

Aging is a complex process that significantly contributes to age-related diseases and poses significant challenges for effective interventions, with few holistic anti-aging approaches successfully reversing its signs. Heterochronic parabiosis studies illuminated the potential for rejuvenation through blood-borne factors, yet the specific drivers including underlying mechanisms remain largely unknown and until today insights have not been successfully translated to humans. In this study, we were able to recreate rejuvenation of the human skin via systemic factors using a microphysiological system including a 3D skin model and a 3D bone marrow model. Addition of young human serum in comparison to aged human serum resulted in an improvement of proliferation and a reduction of the biological age as measured by methylation-based age clocks in the skin tissue. Interestingly, this effect was only visible in the presence of bone marrow-derived cells. Further investigation of the bone marrow model revealed changes in the cell population in response to young versus aged human serum treatment. Using proteome analysis, we identified 55 potential systemic rejuvenating proteins produced by bone marrow-derived cells. For seven of these proteins, we were able to verify a rejuvenating effect on human skin cells using hallmarks of aging assays, supporting their role as systemic factors rejuvenating human skin tissue.

## INTRODUCTION

Aging is associated with a gradual decline of functional tissue, which contributes to an increased susceptibility to diseases and death during adulthood. As the population’s life expectancy increases, it has become a major interest to understand the mechanisms of aging with the aim to promote healthy aging and extend the disease-free lifespan. The skin, as our largest organ, is a valuable tissue to investigate aging, as first signs of aging are mostly visible, and it reflects the overall human health [1].

To date, only few approaches have been demonstrated to holistically rejuvenate tissues by simultaneously reversing multiple hallmarks of aging. Among them is heterochronic parabiosis, a unique approach of joining blood circulation systems of two animals of different ages [2]. Old mice exposed to a juvenile circulation exhibited signs of tissue rejuvenation, i.e., the function and cellular activity of the organs and tissues were improved. Furthermore, intravenous injections of the exosome fraction of young blood plasma from piglets to old rats showed a significant reduction of the epigenetic age in the blood, heart, and liver [3]. For the skin, it has been demonstrated that parabiosis improved tissue structure, led to less senescent cells and an altered gene expression pattern. [4]. Unlike in other organs, the influence of systemic rejuvenation interventions on the skin’s biological age have not yet been investigated. Epigenetic changes represent a reversible primary hallmark of aging, with DNA methylation-based age clocks serving as well-accepted multi-tissue biomarker for assessing aging and rejuvenation [5]. Epigenetic age clocks are sophisticated computational models, that utilize DNA methylation patterns to accurately estimate the biological and functional age of an individual or organ [6]. Trained on empirical DNA methylation data from various tissues, such as skin [7] or blood [8], across diverse age groups, these models can reliably predict the biological age of unknown samples. Recently, a human clinical phase I study measured a reduction of the biological age of the blood and improved biomarkers after intramuscular injection of umbilical cord plasma concentrate into elderly study participants, giving first evidence that rejuvenation by systemic factors might also be observed in humans [9]. However, how systemic factors influence human organs other than the blood, such as the skin, is still unknown. Moreover, so far only few specific drivers of systemic aging and rejuvenation including the underlying mechanism have been identified. For example, heterochronic parabiosis experiments identified tissue inhibitor of metalloproteinases 2 (TIMP-2), granulocyte-monocyte-colony stimulating factor (GM-CSF), growth differentiation factor 11 (GDF-11) and thrombospondin-4 (THBS4) together with the secreted protein acidic and rich in cysteine-like 1 protein (SPARCL1) to improve several age-associated degenerative processes in the muscle and brain [10, 11]. Furthermore, GDF-11 has been demonstrated to have rejuvenating effects on the human skin tissue by increasing procollagen 1 and hyaluronic acid production, accelerating proliferation and changing gene expression *in vitro* and *ex vivo* [12, 13]. However, except for THBS4, SPARCL1 and GDF-11, most factors identified to date were only validated within rodent models, likely due to challenges faced when transferring insights gained within animal models to human cell culture systems.

Microphysiological systems (MPS), also known as organ-on-a-chip platforms, offer an innovative way to study human biological processes and to recapitulate specific elements of the *in vivo* conditions by using organoids comprised of multiple human cell types and by integrating a recirculating media flow for mimicry of the blood circulation [14]. A first engineered MPS system for the identification of rejuvenating factors of murine muscles has been reported allowing to partially mimic parabiosis *in vitro* [15], but there is still a lack of systems investigating human aging.

In this study, we used an MPS to translate the rejuvenation approach of heterochronic parabiosis experiments to an *in vitro* cell culture system in order to investigate human skin aging and rejuvenation systemically as well as to elucidate the molecular mechanisms driving age-related changes in the skin [16].

Beside systemic factors, the blood comprises also a cellular fraction, originating from the bone marrow (BM) niche. The BM harbors a heterogenous population of cells including hematopoietic stem cells (HSCs), mesenchymal stem cells (MSCs), and various lineage committed progenitor cells. HSCs are responsible for the lifelong production of all blood cell lineages through a tightly regulated process of self-renewal and differentiation. The BM-derived cells are known to be strongly influenced by their environment and to secrete different molecules in response to the blood circulation including cytokines, growth factors and chemokines which play critical roles in regulating hematopoiesis, immune responses, and tissue repair [17]. HSCs and all BM-derived immune cells, along with their bioactive secretome, significantly influence skin physiology, with research demonstrating that BM stem cells are mobilized to migrate to both wounded and non-wounded skin and play a critical role in skin regeneration, repair, and homeostasis [18, 19]. Aging-related changes of the BM-niche are widely studied and described as a reduction in the self-renewal capacity and functionality of HSCs, alterations in the microenvironment including impaired MSCs, a decline of lymphoid cell populations, inflammaging and epigenetic changes [20]. The accumulation of pro-inflammatory cytokines by aged BM-derived cells, drives the skin aging process leading to skin senescence, while impaired HSCs fail to accurately facilitate wound healing resulting in reduced regeneration capacity and weakened barrier integrity of aged skin [21]. For this reason, we decided to combine a full thickness skin model with an established BM model [22] in a multi-organ chip system. After the successful co-culture, human serum from young and old donors was introduced into the system to mimic the effects observed in heterochronic parabiosis experiments. We could show that young human serum altered BM differentiation and improved the skin’s biological age, morphology, and cellular proliferation. Interestingly, effects were only visible when combining both organoids in one system, underlining the importance of cells present in the circulation as mediators of the systemic organ-crosstalk and multi-organ MPS as technological prerequisite to study systemic rejuvenation. Consequently, rejuvenating effects on the skin seemed to be the result of different factors secreted by the BM in response to young circulating blood factors. We found 55 age-relevant proteins produced by the BM model treated with young vs. old human serum, including proteins for which we were able to verify improvement of aging hallmarks in human skin cells *in vitro*. Our study gives first proof of rejuvenating effects of systemic factors on human skin and identifies seven proteins as important drivers in the skin rejuvenation process.

## RESULTS

### Young human serum alone does not improve aging markers of human skin models

Based on the findings from heterochronic parabiosis experiments, we hypothesized that the addition of either young (aged <30 years) or old (aged >60 years) human serum to a human skin model should rejuvenate or age the tissue.

**Figure 1:**
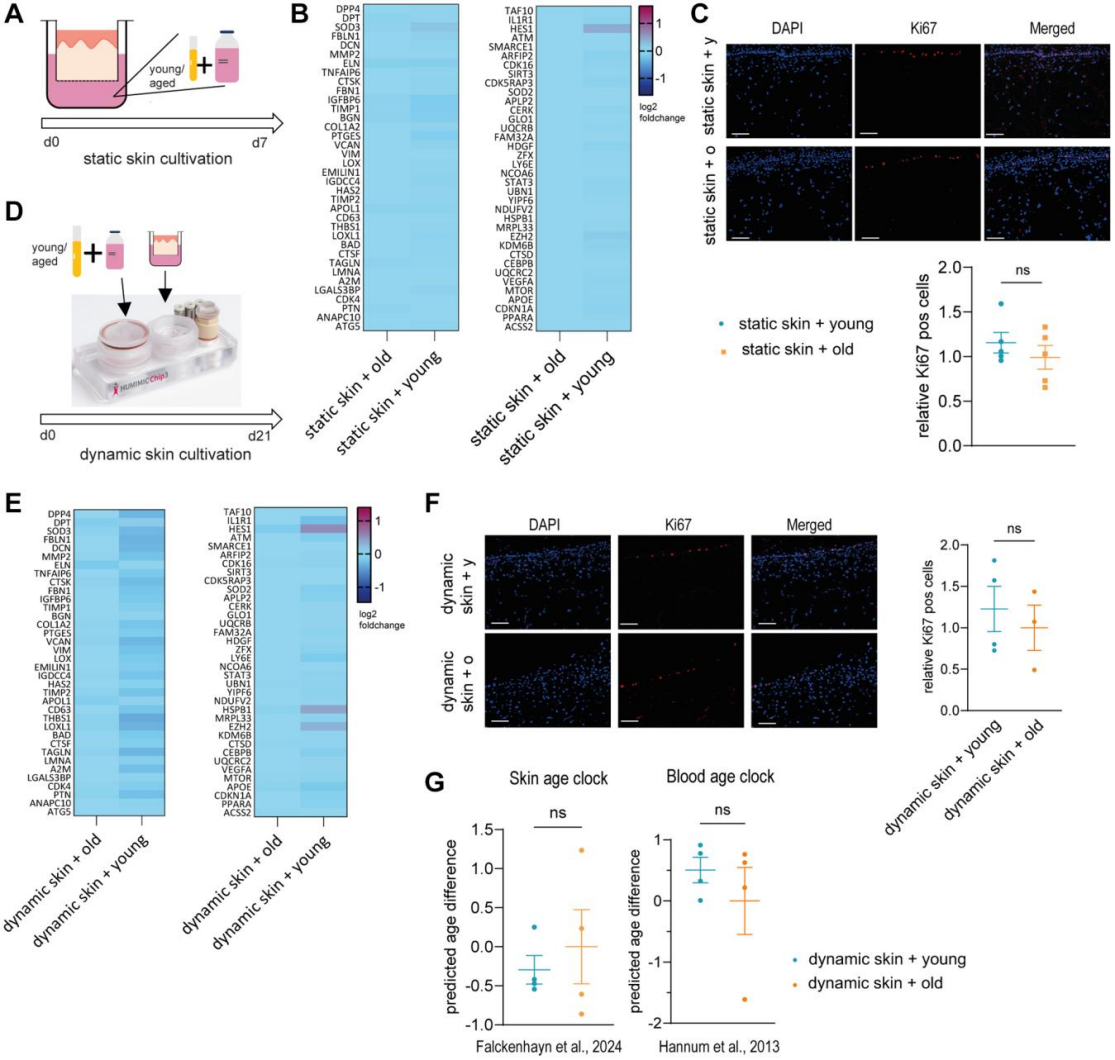
**Young or old human serum alone does not have an effect on 3D skin models in static or dynamic culture.** (**A**–**C**) Human 3D skin models (Phenion^®^) were statically cultured with either young (<30 years) or old (>60 years) human serum as depicted in (**A**) for 7 days before analysis. (**B**) Heatmap indicating relative gene expression of skin models treated with old vs. young human serum, normalized to treatment with old serum. (**C**) Cryosections of treated skin models were analyzed by immunofluorescence staining of Ki67 (red). Representative images (scale bar = 100 μm) are shown in the upper panel. Bar graphs show the relative proportion of Ki67+ cells normalized to treatment with old serum. (**D**–**F**) Human long life 3D skin models (Phenion^®^) were cultured dynamically using the HUMIMIC Chip3plus for 21 days in the presence of young or old human serum as depicted in (**D**). (**E**) Heatmap showing relative gene expression of dynamically cultured 3D skin models treated with old vs. young human serum, normalized to the control cultured with old serum. (**F**) Immunofluorescence staining of Ki67 (red) of dynamic 3D skin models comparing old to young human serum. Representative images (scale bar = 100 μm) are shown. Bar graphs show the relative proportion of Ki67+ cells normalized to treatment with old serum. (**G**) Determination of the DNA methylation-based biological age using the skin DNA methylation clock [7] and the blood age clock [8], normalized to treatment with old serum. Data are shown as mean values +/*−* SEM obtained from 1 experiment with 3–5 replicates, unpaired *t*-test, ns = *p* > 0.05, Author of HUMIMIC Chip3plus image in (**D**): TissUse GmbH, licensed under CC BY ND 4.0.

To validate this hypothesis, we statically cultivated Phenion^®^ full-thickness insert skin models supplemented with 10% human young (mix of 10 donors aged <30 years) or old (mix of 10 donors aged >60 years) serum for seven days (Figure 1A). First, we compared gene expression of treated skin models analyzing a panel of genes expressed in the dermis which have been associated with aging, such as *DPT, DCN, THBS1* and EZH2 (Supplementary Table 1). However, the gene expression profile of the skin models cultivated with young serum was not different from the models cultivated with old serum (Figure 1B).

We then also investigated the effects of serum on skin models on a histological level, examining proliferation by comparing the percentage of Ki67 positive cells. The regenerative capacity is an important aging biomarker, as it was shown that the amount of Ki67 positive cells declines in aged skin [23]. Again, there was no significant difference between the cultivation with young and old human serum (Figure 1C).

To exclude that the cultivation with young or old human serum was too short to observe visible effects, we extended the cultivation time from 7 days to 21 days and incorporated the skin model into an MPS. We used the HUMIMIC Chip3plus from TissUse to enable a longer cultivation period and to emulate a more physiological environment including fluid shear stress [24]. In this approach, Phenion^®^ full-thickness long life insert skin models were cultivated with 10% human young or old serum (Figure 1D). After three weeks of dynamic cultivation, the gene expression profile of the skin models was analyzed using the same panel as before. Again, no significant difference was detected between the cultivation with young serum and the cultivation with old serum (Figure 1E). Furthermore, the immunofluorescence staining of Ki67 did not show significant differences between the cultivation with young or old human serum (Figure 1F). Lastly, we analyzed the biological age of the skin tissue using our own skin age clock [7] and the well-known blood age clock [8] (Figure 1G). However, we could not detect any significant changes.

Taken together, we could not show a direct rejuvenation effect of young systemic factors on the human skin.

### Establishment of a dynamic co-cultivation of skin model and BM model

As all cells derived from the BM circulate together with systemic factors through the human body and secrete molecules that reach all tissues, we wanted to recreate that environment to investigate systemic aging more accurately. Therefore, we created a co-culture of a BM model including different myeloid immune cells and a skin model within the HUMIMIC Chip3plus.

**Figure 2:**
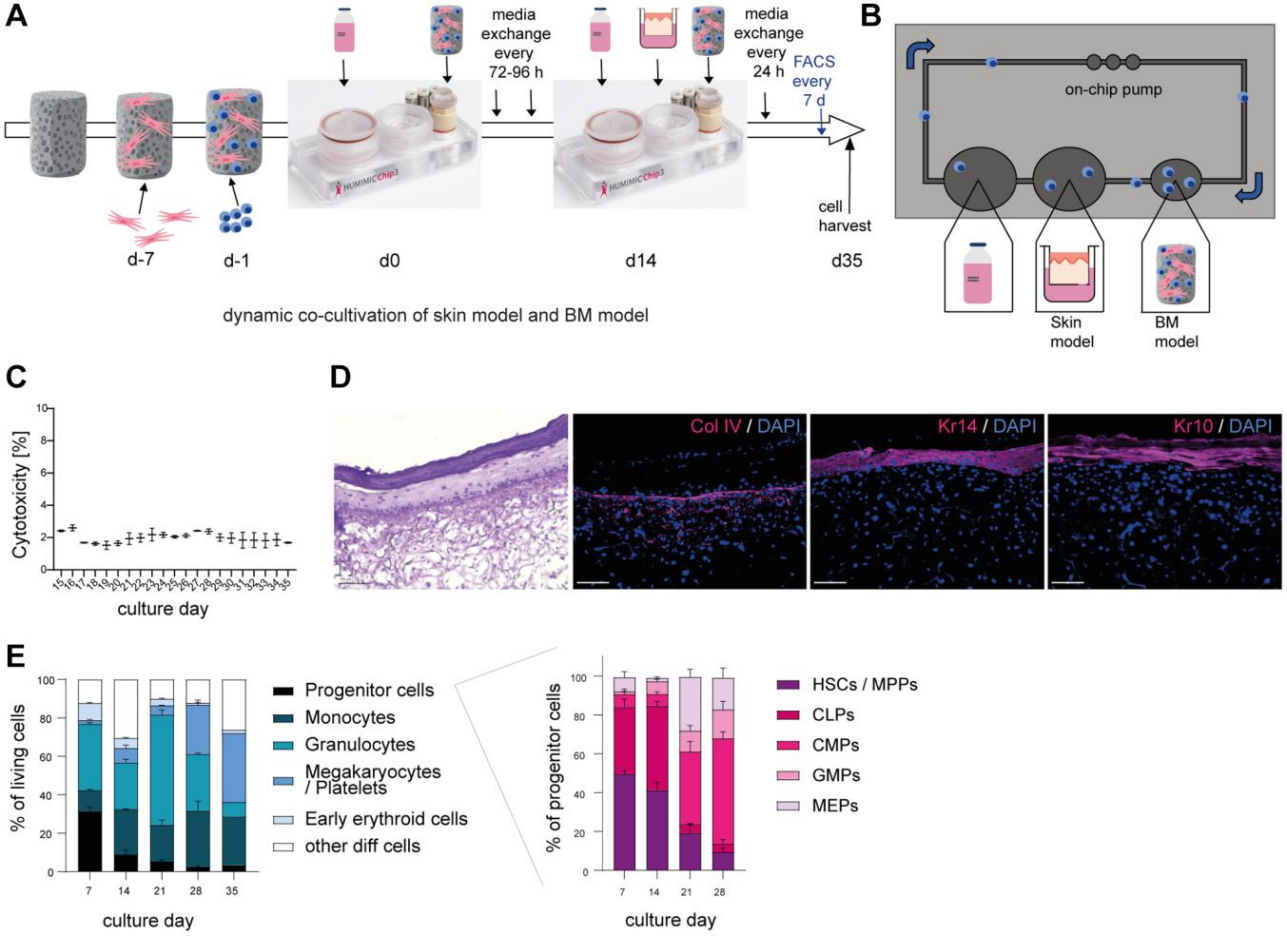
**Successful co-cultivation of skin model and BM model in a long term dynamic *in vitro* MPS.** Human BM-MSCs were pre-cultured on a hydroxyapatite coated zirconiumoxide based Sponceram^®^ scaffold for 7 days, before adding human BM-CD34+ cells and transfer to the HUMIMIC Chip3plus. After two weeks, 3D long life skin models (Phenion^®^) were added to the Chip for another 3 weeks as depicted in (**A**). (**B**) Top view of the HUMIMIC Chip3plus illustrating the composition including skin model, BM model, media flow through the on-chip pump and recirculating BM-derived cells. (**C**) Measurement of LDH release in the supernatant of the co-culture. Cytotoxicity was determined as the percentage of released LDH normalized to the maximum LDH release of the skin models and BM cells after induced lysis. (**D**) Hematoxylin and eosin (left) and immunofluorescence (right) staining of Collagen IV (Col IV, red), Keratin 14 (Kr14, red) and Keratin 10 (Kr10, red) of the 3D skin model. Representative images, scale bar = 100 μm. (**E**) The proportions of different BM cell populations were determined using flow cytometry. Left, the percentage of all BM model populations (progenitor cells, monocytes, granulocytes, platelets/megakaryocytes, and early erythroids) among living cells is shown. Right, the percentage of progenitor cell populations such as hematopoietic stem cells (HSCs) and multipotent progenitors (MPPs), common lymphoid progenitors (CLPs), common myeloid progenitors (CMPs), granulocyte-monocyte progenitors (GMPs) and megakaryocyte-erythrocyte progenitors (MEPs) among all progenitor cells is depicted. Data are shown as mean values +/*−* SEM obtained from 1 experiment with 1–2 replicates. Author of HUMIMIC Chip3plus image in (**A**): TissUse GmbH, licensed under CC BY ND 4.0.

The dynamic BM model was created with small adaptations as first described by Sieber and colleagues [22]. Human BM MSCs were seeded on a hydroxyapatite coated zirconiumoxide based Sponceram^®^ and cultivated to create a human BM niche like environment. Onto this scaffold, human BM CD34+ cells were added and differentiated towards myeloid cells within the HUMIMIC Chip3plus. After two weeks, the co-culture with the Phenion^®^ full-thickness long life inserts skin model started and was kept for another 21 days (Figure 2A). This dynamic cultivation facilitates a continuous flow of media through the on-chip pump and microfluidics, enabling the recirculation of BM-derived blood cells and the exchange of all secreted factors between the skin and BM (Figure 2B). To examine the viability of the co-culture, the release of LDH was measured and the cytotoxicity calculated. The co-culture was viable over the whole cultivation time as determined by a cytotoxicity below 5% (Figure 2C). Viability was maintained only with SFEM II culture medium supplemented with growth factors, as other media resulted in significant loss of viability and differentiation capacity (Supplementary Figure 1). The hematoxylin and eosin staining confirmed a reasonably developed and evenly structured skin model [25], including a dermis, epidermis, and stratum corneum, over the entire co-cultivation time of 21 days (Figure 2D). Staining of three essential skin structure and differentiation markers collagen IV (Col IV), keratin 14 (Kr14) and keratin 10 (Kr10) showed the well-developed basal membrane and verified the presence of both early (Kr14) and late (Kr10) keratinocyte differentiation during the whole course of culture (Figure 2D). Using flow cytometry, the cell composition of BM cells was analyzed over the entire cultivation time of 35 days (Figure 2E and Supplementary Figure 2). After seven days of culture, a quarter of the cell population were progenitor cells, while some monocytes and granulocytes, early erythroid cells as well as a small portion of megakaryocytes and platelets had already developed. Over time, the portion of progenitor cells decreased, while more monocytes and granulocytes developed. Furthermore, the percentage of early erythroid cells was diminished, and the number of megakaryocytes and platelets increased. Hence, all myeloid cells were present within the created BM model and all cell populations could be detected over the entire cultivation time. Among the progenitor cells, HSCs, multipotent progenitors (MPPs) and common lymphoid progenitor cells (CLPs) were decreased over time. Furthermore, common myeloid progenitor cells (CMPs), granulocyte-monocyte progenitor cells (GMPs) and megakaryocyte-erythroid progenitor cells (MEPs) developed at a later stage and constituted the majority of cell populations from culture day 21 on (Figure 2E, right).

Taken together, we successfully established a vital co-culture of a skin model and a BM model including relevant blood cells such as myeloid immune cells and various progenitor cell populations over an extended cultivation period of 21 days.

### Skin and BM model show rejuvenated properties when exposed to young human serum

After the successful establishment of a dynamic co-culture model, we added either 10% young or 10% old human serum to the dynamic co-culture, composed of skin model and BM model cultured in SFEM II supplemented with growth factors, to investigate the impact on skin and BM aging biomarkers.

**Figure 3:**
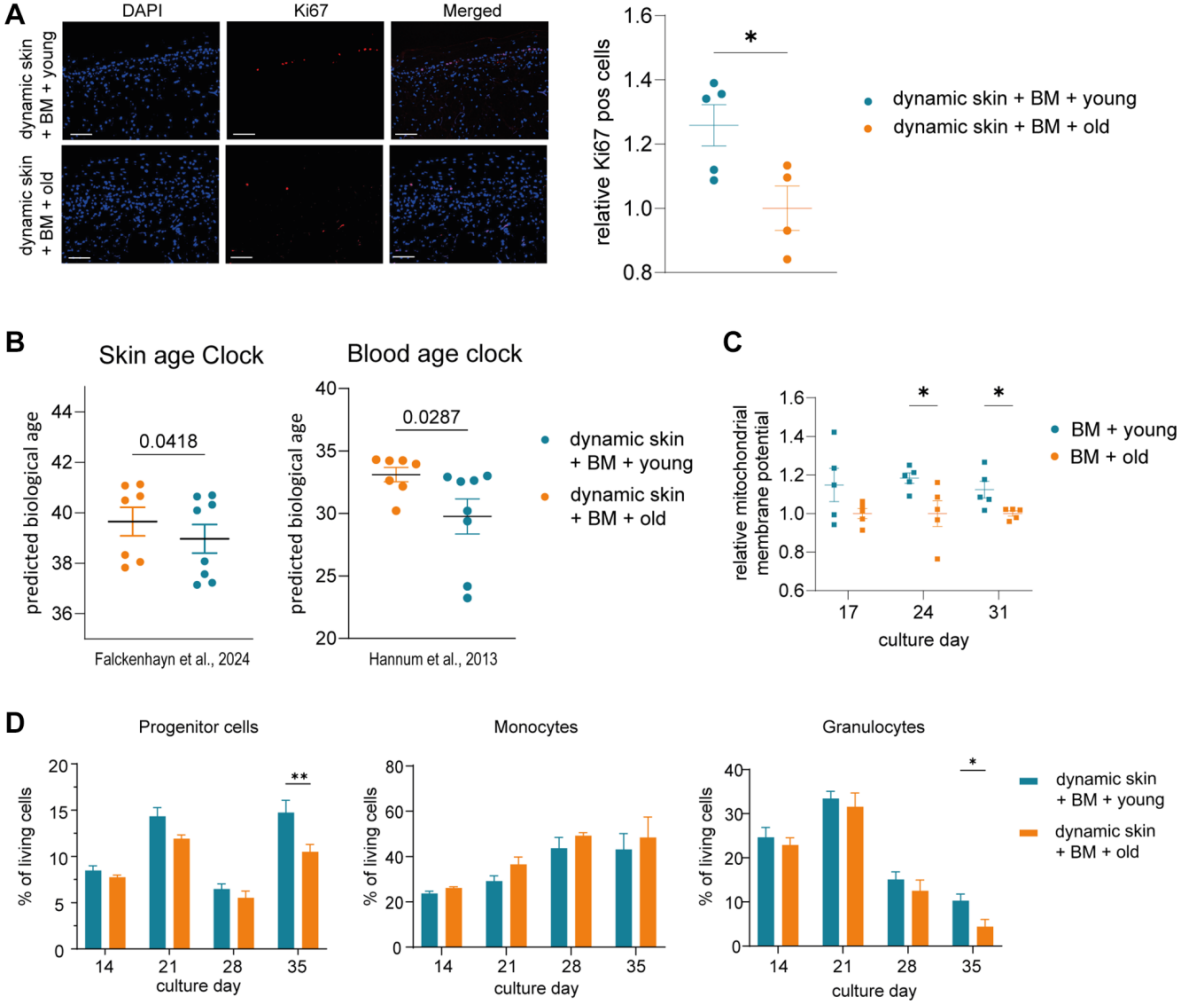
**The skin model and the BM model show rejuvenated properties when co-cultured with young human serum in a long term dynamic *in vitro* MPS.** The BM model was precultured in the HUMIMIC Chip3plus. After two weeks (culture day 14), the 3D long life skin model (Phenion) was added to the system and the co-culture treated with either young or old human serum for three weeks (culture day 14–35). (**A**) Immunofluorescence staining of Ki67 (red) of the 3D skin model. Representative images (scale bar = 100 μm) are shown on the left. Bar graphs show the relative proportion of Ki67+ cells normalized to treatment with old serum. (**B**) Determination of the DNA methylation-based biological age of the 3D skin models using the skin DNA methylation clock [7] and the blood age clock [8]. (**C**) Mitochondrial membrane potential of BM cells harvested on culture day 17, 24 and 31 normalized to treatment with old serum. (**D**) Flow cytometric analysis of BM cells. Bar graphs indicate the proportion of progenitor cells (left), monocytes (middle) and granulocytes (right) of all live BM cells after 14, 21, 28 and 35 days of culture. Data were obtained from one (**A**, **C**) or two (**B**, **D**) experiments with 4–7 replicates, shown as mean values +/*−* SEM (**A**, **C**, **D**) or mean values +/*−* SD (**B**). Unpaired *t*-test (**A**, **B**) or two-way ANOVA with Bonferroni correction (**C**, **D**), ^*^p < 0.05, ^**^p < 0.01.

Interestingly, we detected a significant increase in Ki67 positive cells in the dynamic skin model co-cultured with BM model and young serum compared to the model co-cultured with BM and old serum, indicating an improved regenerative capacity of the tissue (Figure 3A). Furthermore, our skin-specific age clock as well as the applied blood age clock predicted a significantly decreased biological age in skin models cultured with BM and young serum compared to cultures with BM and old serum (Figure 3B). The addition of human serum enhanced the proliferation capacity of human skin models compared to serum-free conditions, most likely through the presence of plentiful growth factors (Supplementary Figure 3).

Analyzing the influence of serum on BM-derived blood cells, the cultivation with young serum resulted in significantly more progenitor cells on culture day 35 (Figure 3D). Already before, a clear but not significant trend of a higher proportion of progenitor cells in the MPS treated with young vs. old serum was visible over the entire co-cultivation time. Moreover, the portion of granulocytes was significantly increased after 35 days of the cultivation with young serum (Figure 3D). In contrast, among the further differentiated cells, the proportion of monocytes was slightly but not significantly decreased in BM treated with young serum.

We also measured the mitochondrial membrane potential of the BM cells as another hallmark of aging [26]. The membrane potential was significantly increased in BM cells cultured with young serum on day 24 and 31 (Figure 3C).

In summary, our results provide first evidence of a rejuvenating effect of young human serum, in comparison to old serum, on human skin using our *in vitro* MPS, but only in the presence of the BM model. The BM model in turn showed a significant increase of progenitor cells together with a significant decline of granulocytes and an improved mitochondrial membrane potential in response to treatment with young human serum.

### The BM model produces age-relevant proteins when exposed to human serum

**Figure 4:**
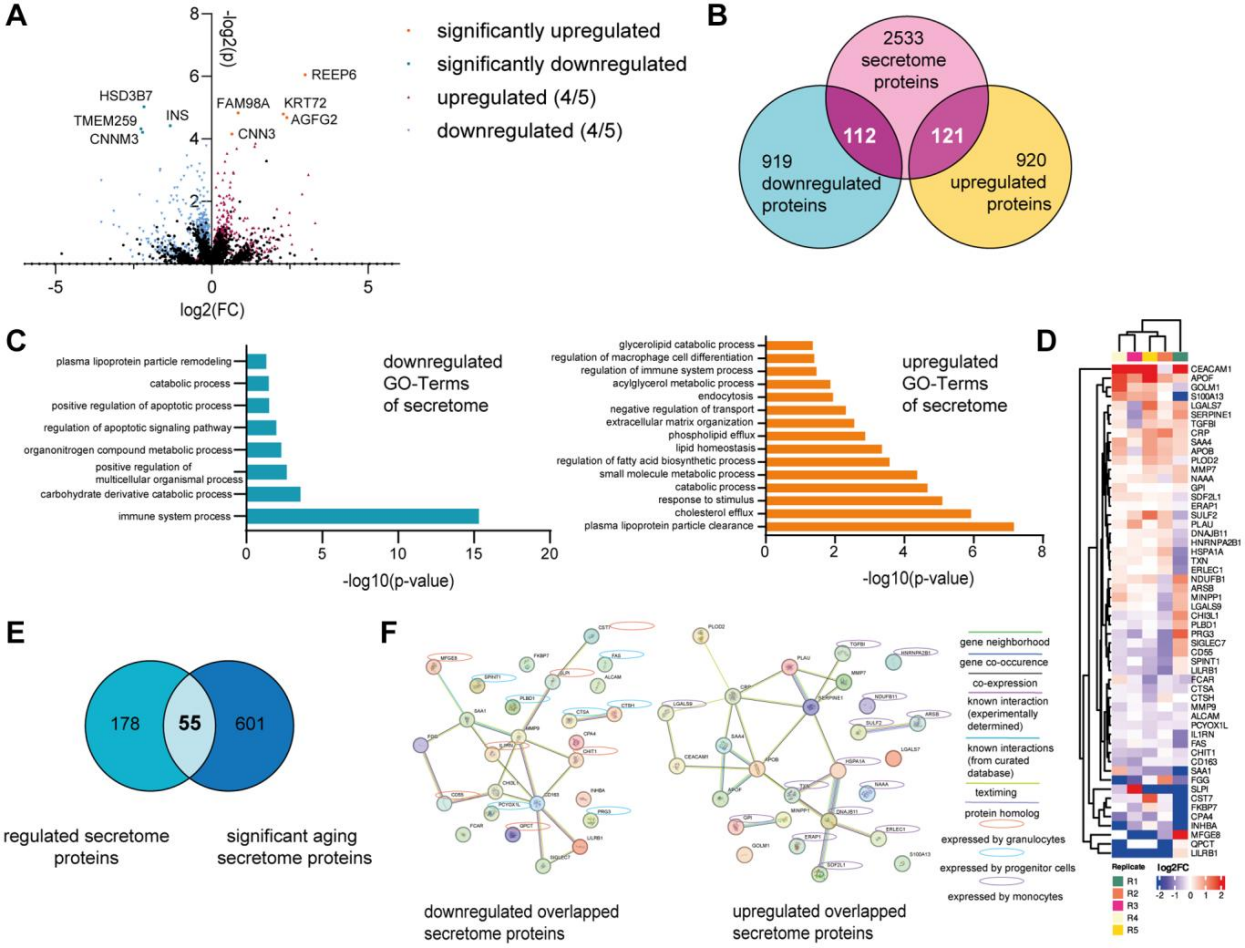
**The BM model secretes age-associated proteins.** The BM model was statically cultured for five weeks. After two weeks, the BM cells were treated with either young or old human serum. On culture day 35, the BM cells were harvested, and the washed cell pellet analyzed using tandem LC-IMS-MS/MS proteomics. (**A**) Log2FC and −log2(*p*-value) of all significantly (*p* < 0.05) up (orange) or downregulated (turquoise) proteins in the BM with old serum compared to young serum. Proteins regulated in the same direction in at least 4 of 5 samples are depicted as well as either upregulated (red) or downregulated (blue). (**B**) Comparison of all regulated proteins to 2772 potentially secreted proteins according to the human protein atlas, creating an overlap of 233 proteins. (**C**) Go-Term analysis of down- (left) and up- (right) regulated overlapped proteins shown in (**B**). (**D**) Heatmap showing the log2FC of the overlapped 55 proteins in (**E**) depicting upregulated (red) and downregulated (blue) proteins with old serum. (**E**) Venn diagram showing the overlap of regulated proteins that belong to the human secretome (left) and secreted proteins that significantly change upon aging (right), resulting in 55 proteins shared between the two categories. (**F**) STRING protein network of the down- (left) and up- (right) regulated proteins from the 55 overlap proteins shown in (**E**). Expression by different BM cell types is highlighted with yellow circles (granulocytes), blue circles (progenitor cells) or violet circles (monocytes). Data were obtained from one experiment with 5 replicates.

In order to identify specific factors produced by the BM model in response to human serum, which could be responsible for rejuvenation effects on the skin model, we performed LC-IMS-MS/MS based proteomics. After 21 days of culture with either young or old human serum (culture day 35), the BM cells were washed with PBS (−/−) to ensure the complete removal of all growth factors and serum components prior to proteomic analysis. Overall, approximately 6,000 different proteins could be detected in all samples. 9 proteins were significantly differentially abundant between BM treated with young and old human serum, among them 5 up- and 4 downregulated proteins (Figure 4A). According to the Human Protein Atlas list “human secretome” (proteinatlas.org) [27], none of these proteins are known to be secreted to potentially impact other tissues. Consequently, we examined in detail all proteins that exhibited regulation in the same direction across a minimum of four out of five samples. If in 4 out of 5 replicates the same trend of abundance (old vs. young) was observed, we considered them as potentially regulated proteins. Using this criterion, we found 2,078 proteins in total, with 1,033 proteins downregulated and 1,045 upregulated in BM models cultured with old compared to young human serum (Figure 4A). We compared those potentially regulated proteins to the 2,772 described secretome proteins from the Human Protein Atlas, creating a list of 233 detected proteins, that could impact the skin as they are secreted to the bloodstream (112 down- and 121 upregulated; Figure 4B). Independent GO-Term overrepresentation analysis of the down- and upregulated human secretome proteins revealed that the 112 downregulated proteins in BM with aged serum led to eight overrepresented pathways. Under these pathways the age-relevant biological processes “cell death and apoptosis,” as well as “lipid metabolism” and “immune system regulation” were identified. In case of potentially upregulated proteins, the GO terms “responses to stress and stimuli,” as well as “transport and triglyceride processes” were overrepresented (Figure 4C). Moreover, we determined the overlap of the potentially regulated secretome proteins with 656 proteins that have already been reported in multiple studies to be significantly age-dependently abundant in the human plasma [28–30]. 55 from our set of 233 potentially regulated secretome proteins have been previously described to be differentially abundant in aged vs. young serum (Figure 4D, 4E). Interestingly, among the 26 potentially downregulated proteins, 14 are expressed by either granulocytes or progenitor cells (according to proteinatlas.org), which could be attributed to the observed significant decrease in cell population after treatment with old human serum. Furthermore, 14 of the 27 potentially upregulated overlapped secretome proteins are expressed by monocytes (according to proteinatlas.org), which increased after the cultivation with old serum. The STRING network analysis of potentially down- and upregulated proteins, showed 13 (down) and 16 (up) potential interconnected proteins based on factors such as co-expression, gene neighborhood or known interactions from databases or experiments (Figure 4F). From this there are five hub proteins identified namely MMP9 and CD163 (down) as well as APOB, CRP and SERPINE1 (up) having at least five potential interconnections to other potential marker proteins.

Taken together, we identified 55 promising age-associated proteins secreted by the BM that might provoke the observed rejuvenating effect on the skin.

### Age-relevant proteins rejuvenate skin tissue characteristics

Since the age-associated proteins secreted by the BM, which were downregulated upon treatment with aged human serum, had the potential to be factors with rejuvenating effects on the skin, we tried to verify their ability to reverse different aging markers. Out of the 26 identified downregulated proteins, 7 were selected based on availability, production in human cells and to include proteins expressed by progenitor cells, granulocytes, and other BM cells. Fibroblasts or keratinocytes isolated from individuals over 60 years of age from a minimum of three and up to twenty different donors were treated with 100 ng/ml of protein for 72 hours [31] and subsequently compared to their untreated control. As a benchmark control, we compared effects to GDF-11 treatment, a systemic factor with reported rejuvenating effects on human skin *in vitro* and *ex vivo*.

**Figure 5:**
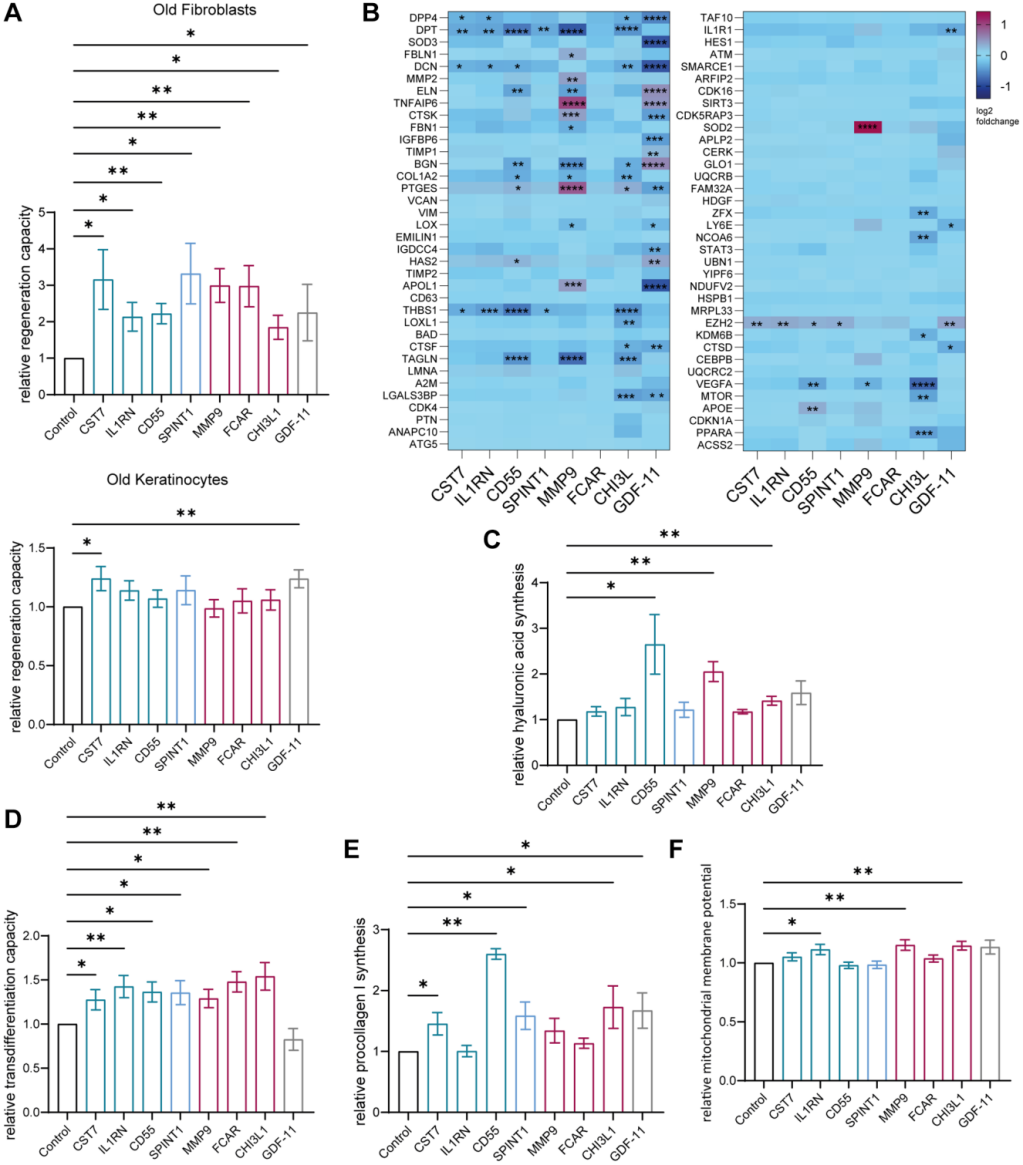
**Age-associated proteins secreted by the young BM model rejuvenate skin cells.** Old (>60 years) human primary dermal fibroblasts and old (>60 years) human primary epidermal keratinocytes were statically cultured and treated with 100 ng/ml of the appropriate downregulated age-associated protein for 72 hours. (**A**) Immunofluorescence staining of Ki67 of fibroblasts (top) and keratinocytes (bottom). Bar graphs show the relative proportion of Ki67+ cells normalized to the corresponding untreated control, *n* = 5–20. (**B**) Heatmap indicating relative gene expression of statically cultured fibroblasts treated with proteins normalized to the untreated control, *n* = 4. (**C**) Bar graph illustrating the relative production of hyaluronic acid normalized to the untreated control, measured in the supernatant of fibroblasts, *n* = 3–7. (**D**) Bar graph showing the relative ability of fibroblasts to differentiate into adipocyte-like cells of fibroblasts treated with proteins normalized to the control cultured without proteins, *n* = 7–14. (**E**) Bar graph showing the relative production of procollagen 1 normalized to the untreated control, measured in the supernatant of fibroblasts, *n* = 3–7. (**F**). Bar graph illustrating the relative mitochondrial membrane potential of treated fibroblasts normalized to the corresponding untreated control, *n* = 6–10. Data are shown as mean values +/− SEM. Paired *t*-test, ^*^p < 0.05, ^**^p < 0.01, ^***^p < 0.001, ^****^p < 0.0001.

The analysis of Ki67 positive cells revealed a significant increase of proliferation of fibroblasts after treatment with cystatin-F (CST7), interleukin 1 receptor antagonist (IL1RN), complement decay-accelerating factor (CD55), Kunitz-type protease inhibitor 1 (SPINT1), matrix metalloproteinase-9 (MMP-9), Fc fragment of IgA receptor (FCAR), chitinase-3-like protein 1 (CHI3L1) and GDF-11 compared to the control without protein. In keratinocytes, treatment with the same proteins did only affect proliferation in response to CST7 and GDF-11 (Figure 5A and Supplementary Table 2). Using the aging panel from before, between three to seventeen genes such as DPT, DCN, THBS1 and TAGLN were significantly differentially expressed after the addition of our newly identified proteins, except for FCAR (Figure 5B). Additionally, investigation of the supernatant of treated fibroblasts revealed significantly increased production of procollagen 1 after treatment with CST7, CD55, SPINT1, CHI3L1 and GDF-11 (Figure 5E and Supplementary Table 2), whereas hyaluronic acid was significantly increased after addition of CD55, MMP9 and CHI3L1 (Figure 5C and Supplementary Table 2). To measure another integrative hallmark of aging, the ability to transdifferentiate into adipocyte-like cells was examined as a readout indicating partial reprogramming of fibroblasts to a phenotype with higher plasticity [32]. After treatment with preadipocyte growth medium for two weeks, all cultures with the seven identified proteins resulted in a significantly higher percentage of adipocyte-like cells compared to the control (Figure 5D and Supplementary Table 2). Finally, the mitochondrial membrane potential was assessed and an improvement after treatment with IL1RN, MMP-9 and CHI3L1 asserted (Figure 5F and Supplementary Table 2).

Consequently, we could show that six proteins were able to alleviate more than two hallmark of aging assays in human dermal fibroblasts, supporting their potential role in the observed BM-derived cell mediated skin rejuvenation upon exposure to young serum.

## DISCUSSION

Heterochronic parabiosis as well as plasma transfer studies have demonstrated the rejuvenating effect of blood factors such as proteins or exosomes on aged organs and tissues in rodents [33], but our knowledge of the impact on human tissues and the responsible factors is still very limited [34]. Here, we show for the first time *in vitro* that young human serum containing several systemic factors promotes rejuvenation processes of the human skin. Of note, our results provide first evidence that systemic factors exert rejuvenating effects on the skin exclusively in the presence of BM-derived cells. We hypothesize that these cells increase the concentration of systemic factors to an effective level in response to young human serum within our experimental setup, thereby influencing skin cells. However, further research is necessary to elucidate the specific roles and mechanisms of BM-derived cells in this context. We could attribute the BM-dependent rejuvenating effect of systemic factors to BM-secreted proteins, a process likely driven by alterations in BM subpopulations in response to young human serum. However, it is important to note that our experimental setup relies on the inclusion of various growth factors to establish a viable BM model. While it is assumed that these growth factors could likewise enhance skin characteristics, their effects are negligible when comparing cultures with growth factors and young human serum to those supplemented with growth factors and old human serum. Our study lays a basis for future therapeutic approaches using systemic factors to reverse signs of aging in the human skin.

Using our complex *in vitro* co-culture system including human BM and skin models, we were able to detect rejuvenation of the skin in response to young human serum as determined by increased proliferation as well as a decreased biological age, a readout holistically measuring tissue rejuvenation. The decline of proliferating cells represents one integrative hallmark of aging [35] and the decrease of the proliferation marker Ki67 has also been demonstrated for aged human skin [23]. Previous studies have demonstrated reduced cell proliferation *in vitro* following exposure to aged animal serum [36], however, other investigations utilizing various serum types or cell lines could not observe similar effects [37]. In our current study, the addition of human serum to human skin models revealed increased cell proliferation possibly resulting from the presence of growth factors (Supplementary Figure 3), with the effect of young human serum being significantly more pronounced than that of old human serum. This suggests a rejuvenating influence attributed to young growth factors, in addition to the identified age-related proteins. Epigenetic clocks measuring the biological age based on the DNA methylation pattern have become the most promising and widely used tool to holistically assess aging [6]. Accordingly, the biological age of several tissues, such as blood and liver, was reported to be drastically reduced after heterochronic parabiosis, but has not yet been described for the skin [38].

In our *in vitro* approach, rejuvenation of skin tissue is dependent on the presence of BM-derived blood cells in the circulation. It has been previously suggested that aged HSCs are not affected by systemic rejuvenation interventions in mice [39]. However, other progenitor cells and progenitor-derived cells were not investigated in this study. In contrast, a more recent study has identified HSCs and further BM progenitor cells as the most susceptible cell types to young blood exposure within murine systems, supporting their important impact on the rejuvenation process [4]. Furthermore, another report described increased proliferation and maintained quiescent state of HSCs in response to systemically released factors [40]. For our human BM model, we were able to show several changes suggesting a rejuvenating effect of young serum. We detected a significant increase in the progenitor cell population in the presence of young human serum, which is in line with the fact that the capacity for self-renewal, an important characteristic of HSCs, diminishes upon aging [41]. In addition, we observed a decrease in the granulocyte cell population after cultivation with old human serum, which has also been described for aged human blood cells [20]. The trend of an increase in the monocyte cell population after cultivation with old human serum is also consistent with the changes observed in aged humans [42]. Moreover, we could show that the young human serum rejuvenated characteristics of the BM by detecting an improved mitochondrial membrane potential of the BM model after the cultivation with young human serum. An impairment of mitochondrial function leading to increased reactive oxygen species production, inflammation and cell death has been described as an antagonistic hallmark of aging [35].

Since in our experimental setup, the skin tissue was only rejuvenated in the presence of BM cells, we were able to investigate responsible proteins by proteome analysis of the BM.

We identified 55 potentially secreted proteins, which were differentially produced by the BM in response to serum treatment and which have already been described to be altered in the human serum upon aging [28–30]. For seven proteins, we were able to show rejuvenating effects by measuring improvements in at least two hallmarks of aging assays. Despite the previous reports of numerous systemic factors that change upon aging in human serum, only a limited number of proteins have been demonstrated to contribute to the systemic rejuvenation process, and their effect was mostly proven in rodent-studies focusing on organs such as the muscle, brain, heart and liver [10]. To our knowledge, GDF-11 is the only factor reported to improve aging characteristics in human skin by regulating progenitor proliferation, protection from inflammatory responses and improving ECM structures [12].

Among the seven proteins with rejuvenating effects on fibroblasts *in vitro*, CHI3L1 improved all six of our tested age markers composed of improved proliferation, altered expression of age-associated genes, increased procollagen 1 and hyaluronic acid synthesis, improved differentiation of fibroblasts towards adipocyte-like cells and elevated mitochondrial membrane potential. Consequently, CHI3L1 was able to improve more hallmarks of aging in comparison to GDF-11, which we used as a benchmark because it has been already described as a skin rejuvenating factor. CHI3L1 is known to enhance cell survival by protecting against apoptosis, promoting cell division, facilitating tissue remodeling, and acting as signaling molecule that mediates inflammatory responses including repair mechanisms, consistent with our detected influence on multiple markers of skin aging. So far, its effects have only been reported in brain and lung tissues [43]. CD55 and MMP-9 improved five of our tested age markers. MMP-9 is known to induce production of SCF leading to recruitment of progenitor cells [44], providing a potential explanation for the observed increase of progenitor cells after treatment with young human serum in our system. Moreover, MMPs play a critical role in the regeneration of skin tissue and the differentiation of the epidermis [45]. CD55 as a complement decay-accelerating factor inhibits complement activation, potentially counteracting an increased level of inflammation known as inflammaging, including increased complement levels associated with aging in the skin [46]. Also, IL1RN and FCAR, which each improved three aging hallmark in the fibroblasts, are both immune regulators that might exert rejuvenating effects mitigating inflammaging. While the role of FCAR in aging is still unclear, increased IL-1 levels are known to promote skin inflammaging and as an IL-1 receptor antagonist, IL1RN may compensate this process [46]. The protease inhibitors CST7 and SPINT1 both improved the same four aging markers proliferation, gene expression, transdifferentiation capacity and procollagen 1 synthesis. The two proteins are known to be involved in cellular senescence and the degradation of collagen and elastin, suggesting a possible mechanism for their observed rejuvenating effect on skin cells [47]. Interestingly, they are both secreted by granulocytes, and SPINT1 mostly by progenitor cells, which were the two cell populations among the BM cells we showed to be increased after adding young human serum. It is important to highlight that we intentionally chose to investigate the specific effects of various factors on individual 2D cell cultures, allowing to elucidate cellular mechanisms and responses that may be obscured in more complex 3D skin models. By focusing on 2D cell cultures we could explore multiple hallmarks of skin aging and identify critical signaling pathways and molecular interactions that contribute to the overarching biological processes involved, while early gene expression responses may also be significant and need further exploration. The primary focus of this study was the identification of key age-related proteins that may rejuvenate the human skin tissue, however, given the documented negative effects of old serum on cellular function in different experimental setups [36], future research should investigate the aged secretome of BM-derived cells to elucidate the causal mechanisms and identify critical aging regulators that contribute to the detrimental effects of old serum on skin cells.

In conclusion, using our *in vitro* MPS system including BM and skin, we were able to reproduce systemic rejuvenating effects of circulating blood factors on the human skin, which have been so far only demonstrated in rodent heterochronic parabiosis studies. Moreover, we identified several proteins that might be responsible factors to rejuvenate the skin in our system. However, systemic factors not only include proteins, but also e.g. exosomes, metabolites and microRNAs [48]. Future studies are needed to further validate our identified proteins in the context of systemic skin rejuvenation and aging, for example the usage of aged cells within 3D skin models or the prolongation of the culture time would be interesting to explore [49]. Furthermore, it will be interesting to examine the systemic rejuvenation on the skin in combination with other organs such as for example the kidney as it plays an important role as filtrating organ in systemic aging [50].

## METHODS

More and detailed methods can be found in Supplementary Materials and Methods.

### Skin model culture

The Phenion^®^ full-thickness insert skin models were cultured within a transwell in air-liquid interface culture medium (Phenion) supplemented with 10% human young (mix of 10 donors aged <30 years) or old (mix of 10 donors aged >60 years) serum (Zen-Bio, Supplementary Table 3) at 37°C with 5% CO_2_. The medium was exchanged every day, and the culture kept for 7 days during the static cultivation. For dynamic cultivation within the HUMIMIC Chip3plus (TissUse GmbH), the circuit was filled with 2 mL fresh medium, and the skin models were added to the middle 24-well compartment of the chip. The system was then connected to the HUMIMIC Starter control unit and operated at a pressure and vacuum of 500 mbar with 0.5 Hz as the pump frequency. Half of the medium was exchanged daily, and the culture kept for 21 days. We utilized standard Phenion^®^ skin models instead of Phenion^®^ aged skin models to avoid potential unphysiological responses of the skin cells due to the drug-induced aging process as well as to increase the chance to identify early changes preceding visible signs of aging.

### BM culture

The BM model was constructed and cultivated as described previously [22]. Briefly, 500.000 precultured human BM-MSCs (Lonza Group AG) were seeded onto a hydroxyapatite coated zirconiumoxide based Sponceram^®^ cylinder (TissUse GmbH). After cultivation for 7 days in DMEM high glucose (Gibco) with 10% FCS, 1% P/S and 1% GlutaMAX (all from Thermo Fisher Scientific), 10.000 human BM CD34+ cells (Lonza Group AG) were added to the system and the medium changed to StemSpan™ SFEM II (Stemcell Technologies) + 50 ng/mL SCF + 10 ng/mL TPO + 100 ng/mL Flt3-L (all from Peprotech) + 1% P/S. The next morning, the cultivated Sponceram^®^ cylinders were transferred into a 24-well plate and the medium exchange every two to three days.

For dynamic cultivation on the HUMIMIC Chip3plus, the preincubated scaffolds were transferred to the 96-well compartment. The circuit was filled with 2 mL fresh medium, and the system connected to the HUMIMIC Starter control unit. The HUMIMIC Chip3plus was operated as described above. During the first two weeks, 1.6 mL of medium was exchanged twice a week. Once a week, the BM cells were sampled through resuspension during the medium exchange for further flow cytometry analysis.

### Co-culture of the BM and skin model within the HUMIMIC Chip3plus

After 14 days of dynamic BM model culture, the Phenion^®^ full-thickness long life insert skin models were added to the middle 24-well compartment of the HUMIMIC Chip3plus and the medium changed to StemSpan™ SFEM II (Stemcell Technologies) + 50 ng/mL SCF + 10 ng/mL TPO + 100 ng/mL Flt3-L + 1 ng/mL M-CSF + 1 ng/mL GM-CSF (all from Peprotech) + 1% P/S (Thermo Fisher Scientific) + 10% human young or old serum. The co-culture was kept for further 21 days and 0.6 mL of the medium of medium exchanged daily. Additionally, BM cells were collected once a week and analyzed by flow cytometry.

After 3 weeks of co-culture, the skin model was removed from the HUMIMIC Chip3plus and cut in half for further analysis. For histological analyses, one part of the skin model was directly frozen within Tissue-Tek^®^ (Sakura Fintek) in liquid nitrogen. For RNA and DNA analyses, the epidermis and dermis were separated using tweezers and frozen separately in liquid nitrogen.

### Flow cytometry

Harvested single cell suspension of BM cells from the HUMIMIC Chip3plus were resuspended in FcR blocking reagent (1:12.5, Miltenyi Biotech) and incubated for 5 min on ice. Afterwards, different combinations of the following antibodies were added (1:50) and incubated for another 20 min in the dark on ice: anti- CD123-PE, CD15-PE-Cy7, CD38-PerCP-Cy5.5, CD-45RA-BV421, CD16-APC, CD14-BV711, CD34-APC-Cy7, CD13-BV711, CD41-PE-Cy7, CD71-PerCP-Cy5.5, CD36-BV421, CD235a-APC, CD229-PE (all BioLegend, Supplementary Table 4). To discriminate dead cells, the Zombie Green fixable viability kit (BioLegend) was used according to manufacturer’s instructions. Cells were measured at the flow cytometer (BD LSRFortessa™ Cell Analyzer, BD Biosciences). Data were analyzed using FlowJo v10.7.1 software.

### nCounter XT gene expression analysis

Frozen dermis and epidermis samples were thawed in 350 μL RLT lysis buffer (Qiagen), mixed with 1% ß-mercaptoethanol (Sigma-Aldrich) and homogenized using ceramic beads and the Precellys 24 homogenizer (Bertin Technologies). The RNA was isolated according to protocol using the RNeasy^®^ Mini Kit (Qiagen) and the QiaCube Connect (Qiagen). For gene expression analysis, 70 ng of RNA was used and hybridized using target-specific reporter and capture probes for 24 hours at 65°C. The samples were measured using the nCounter SPRINT profiler system (Bruker) and analyzed with the nSolver software. Normalization was done to control samples and the following housekeeping genes: *GAPDH*, *GUSB*, *OAZ1*, *PUM1* and *UBC*.

### Immunofluorescence staining

For every skin model, four samples of distant 5 μm cryosections were fixed with 4% formaldehyde (Merck), blocked with 3% bovine serum albumin (Miltenyi) and permeabilized using 0.05% Tween^®^ 20 (Sigma-Aldrich). The samples were incubated with the primary antibody (Ki67, Col IV, Kr10 or Kr14, 1:300, Abcam, Supplementary Table 5) overnight, the fluorescent dye-conjugated secondary antibody (1:1000, Invitrogen, Alexa Fluor 546 goat anti-mouse IgG) was added and the ProLong™ Gold Antifade Mountant with DNA Stain DAPI (Invitrogen) used to mount the samples. Pictures were taken at BZ-X810 Keyence microscope and all distant sections analyzed using ImageJ [51]. To calculate the relative Ki67 pos cells, the percentage of Ki67 positive cells of treated skin models was normalized to the percentage of all ski models treated with old human serum.

### DNA methylation analysis

Frozen skin model samples were thawed in PBS (−/−), homogenized using the Precellys 24 homogenizer (Bertin Technologies) and ceramic beads. DNA was isolated using the QIAamp DNA Investigator Kit (Qiagen) according to the manufacturer’s protocol. The quantity of DNA was determined using the Pico green Kit (Thermo Fisher Scientific) before analysis on the microarray Infinium MethylationEPIC v2.0 BeadChip (Illumina), measuring around 935.000 CpG loci. Methylation data analysis was conducted using the R package minfi v1.48.0 [52]. Specifically, raw .idat files were loaded, filtered, preprocessed and quantile normalized by minfi with default parameters. Age clocks are AI-based models which have been trained on several methylation data sets to predict the biological age. To determine the biological age of our skin model, we used the trained AI-models of the skin DNA methylation clock, trained with 378 skin samples [7], and the blood age clock, trained with 656 blood samples [8], given our quantile normalized methylation data from our experiments as input. Epigenetic age clocks have been demonstrated to be applicable for *in vivo* as well as *in vitro* experiments [53], which justified the usage in our experimental set up especially to determine relative changes between different culture conditions.

### LC-IMS-MS/MS proteomics

The proteins produced by BM models cultured with young or old human serum were analyzed. Cell lysates were prepared from 50,000 cells using an 8 M thiourea buffer, the protein concentration determined using the Invitrogen™ Qubit™ Protein Assay Kit and the subsequent protein digestion and peptide purification conducted in accordance with the filter-aided sample preparation protocol [54], including reduction, alkylation, and dual enzymatic digestion with rLys-C and trypsin. The desalted peptides were then evaporated and resuspended in 0.1% formic acid in preparation for analysis. A total of 400 ng of peptides were separated via nano-uHPLC and analyzed on a timsTOF Pro 2 mass spectrometer using DIA-PASEF, which was optimized with py_diAID [55]. The raw data were processed with DIA-NN (version 1.8.1; [56] and a human reference proteome for library-free analysis. The assessment of significant differential abundant proteins was identified using DESeq2 [57], threshold adj. *p*-value <0.05). Data visualization employed R libraries, including dplyr and ComplexHeatmap [58].

### Statistics

If not indicated otherwise all statistical analyses were performed using GraphPad Prism v10.1.0. When comparing two datasets, a Two-tailed unpaired Welch *t*-test was performed. Multiple datasets with repeated measures were analyzed using multiple test correction for adjusted *p*-values via Bonferroni-Test. For analysis of the nCounter gene expression data, multiple unpaired *t*-tests with Bonferroni correction were performed. Data are represented as mean ± standard errors of the mean (SEMs), not significant (ns) *p* > 0.05, ^*^p < 0.05, ^**^p < 0.01, ^***^p < 0.001, ^****^p < 0.0001.

### Data availability statement

The mass spectrometry proteomics data have been deposited to the ProteomeXchange Consortium via the PRIDE [59] partner repository with the dataset identifier PXD059082. All other data parts of this study are available from the corresponding authors on reasonable request.

## Supplementary Materials

Supplementary Materials

Supplementary Figures

Supplementary Tables 1 and 3-5

Supplementary Table 2
