# Supplementary material for: Systemic factors in young human serum influence in vitro responses of human skin and bone marrow-derived blood cells in a microphysiological co-culture system
Source: Aging (Albany NY). 2025 Jul 25;17(7):1784–809. doi: 10.18632/aging.206288 (PMC12339024; doi:10.18632/aging.206288)
Supplement: Supplementary Materials [file aging-17-206288-s001.pdf]

SUPPLEMENTARY MATERIALS

Supplementary Materials and Methods

Skin cell culture

Old (donor age >60 years) human primary dermal fibroblasts and keratinocytes were isolated from human skin samples as previously described [1]. Old fibroblasts were seeded in DMEM supplemented with 10°% human old serum and 1% P/S, while old keratinocytes were seeded in KGM Gold medium (Lonza) at 37°C with 5% CO_2. After an initial incubation overnight, the cells were treated with 100 ng/ml of either CST7, IL1RN, CD55, SPINT1, MMP9, FCAR, CHI3L1 or GDF-11 for 72 hours.

Cytotoxicity analysis

To measure the release of LDH in the supernatant of the MPS co-culture, the Cytotoxicity Detection Kit^{PLUS} (LDH) (Roche) was used according to the manufacturer's instructions. Cytotoxicity was calculated with the following equation:

Cytotoxicity (%) = (measured value – medium control)/(high control – medium control) × 100.

Mitochondrial membrane potential

The JC-10 Mitochondrial Membrane Potential Assay Kit (Abcam) was used according to manufacturer's instructions to determine the mitochondrial membrane potential of BM cells cultured with either young or old human serum.

Transdifferentiation capacity assay

72 h treated old (donor age >60 years) human primary dermal fibroblasts were further cultivated for 14 days in PGM-2™ preadipocyte growth medium-2 bulletkit (Lonza) with medium exchange twice a week. The capacity to transdifferentiate into adipocyte-like cells was investigated through lipid staining with HCS LipidTox® Dye red reagent (Thermo Fisher) after nuclei staining with Hoechst 33342 (Thermo Fisher) and fixation in 3.7% formaldehyde. Image acquisition and quantification were performed using the ImageXpress® Pico automated cell imaging system (Molecular Devices).

LC-IMS-MS/MS proteomics

To determine the proteins produced by the BM model cultured with young or old human serum, 50,000 cells were sampled, washed twice with PBS (−/−) and the cell pellet lysed with 200 µL 8 M thiourea buffer and incubated for 5 minutes at 95°C, afterwards cooled on ice. DNA disruption was performed in an ultrasonic bath for 3 minutes, followed by centrifugation for 5 minutes at 16000 × g at 20°C. The supernatant was collected and the protein concentration determined using the Invitrogen™ Qubit™ Protein Assay Kit according to manufacturer's instruction.

The filter-aided sample preparation protocol (modified according to [2]) was used for protein digestion and peptide purification using 15 µg protein. Sample preparation was performed, including reduction, alkylation and rebuffering of the samples. Protein digestion (separate double digestion) and peptide recovery were performed with 10 µl 8 M urea buffer + 40 µl rLys-C digestion solution (1:25 enzyme/protein ratio with a urea concentration <2 M) for 60 min, followed by 40 µl trypsin digestion solution with a 1:25 enzyme/protein ratio and a urea concentration <1 M. The samples were desalted using an Oasis HLB 96-well µElution Plate and eluted using 70% acetonitrile. The tryptic peptides were completely evaporated using a Vacufuge Vacuum Concentrator (Eppendorf) at 30°C for 2 hours and stored at 4°C until peptide concentration determination.

The tryptic peptides were resuspended in 0.1% formic acid and the peptide concentration was determined using a NanoDrop Microvolume Spectrophotometer (Thermo Fisher Scientific). The volume was adjusted with 0.1% formic acid to gain harmonized peptide concentrations and the peptides were analyzed using an ion mobility tandem mass spectrometer (timsTOF Pro 2, Bruker Daltonics) coupled to a nano-ESI source (CaptiveSpray ion source, Bruker Daltonics) and a nano-uHPLC system (nanoElute 2, Bruker Daltonics). A total of 400 ng of peptides were injected onto a trap cartridge (5 mm C18 Trap Cartridge, Thermo Fisher Scientific). Peptide separation was performed over 60 minutes using a multistep gradient from 2% to 35% acetonitrile in 0.1% formic acid on a PepSep XTREME analytical column (25 cm × 150 µm × 1.5 µm, Bruker Daltonics) at a constant flow rate of 500 nl/min. Eluting peptides were ionized at 1.6 kV. The mass spectrometer operated in positive ion mode with data-independent acquisition and parallel accumulation-serial fragmentation (DIA-PASEF). MS1 scans were acquired at 100–1,700 m/z. MS2 scans were accumulated for 80 ms. TIMS range was set to 0.7–1.3 Vs/cm^2. py_diAID was used for optimization of isolation windows [3]. MS2 scans covered a mass range of 300–1,200 m/z comprising 16 DIA-PASEF scans, three ion mobility windows per DIA-PASEF scan and a mean window size of 19 Th. Collision energy ranges from 20 eV to 59 eV depending on the ion mobility of analyzed peptides.

The acquired raw data were analyzed using DIA-NN (version 1.8.1) in library-free mode [4]. A human reference proteome (Uniprot, version 2023_05, reviewed/SWISS-Prot entries only, 20,418 sequences) was used for *in silico* spectral library generation. Precursor and fragment ion accuracy was set to 15 ppm. Heuristic protein inference was disabled, whereas match between runs was enabled. All other parameters were set as default.

DAP analysis DIA-NN output was preprocessed by changing the measured intensities to integer values. These values were used as input in DESeq2 ([5], version 1.34.0) or for manual calculation of log2 fold changes (L2FC). As experimental design we used BM+young serum (n = 5) versus BM+old serum (n = 5). To identify significantly abundant proteins we used the adjusted p-value below 0.05 as threshold. For the identification of potentially regulated proteins we used the following criteria: if in 4 out of 5 replicates the same trend of the manually calculated L2FC was positive (UP: BM+old serum vs. BM+young serum) or negative (DOWN: BM+young serum vs. BM+old serum) we considered them as potentially regulated proteins. For general data processing the R library dyplr ([6],1.1.4. https://CRAN.R-project.org/package=dplyr) tidyverse (https://doi.org/10.21105/joss.01686; version 2.0.0) and data.table ([7], version 1.15.2. https://CRAN.R-project.org/package=data.table) were used and for heatmap visualization we used the R library ComplexHeatmap ([8] ,version 2.10.0).

To further examine the identified proteins, a functional enrichment analysis using g:Profiler (version e111_eg58_p18_f463989d) with g:SCS multiple testing correction method applying significance threshold of 0.05 [9] and a STRING (version 12.0) network analysis [10] of the either down- or upregulated proteins was performed.

Supernatant analysis

The Procollagen type I C-Peptide (PIP) EIA Kit (Takara) and the Corgenix Hyaluronic Acid ELISA (Orgentec) were used according to manufacturer's instructions to determine the amount of Procollagen type I and hyaluronic acid in the supernatant of the treated human primary dermal fibroblasts.

SUPPLEMENTARY REFERENCES

1. Gather L, Nath N, Falckenhayn C, Oterino-Sogo S, Bosch T, Wenck H, Winnefeld M, Grönniger E, Simm S, Siracusa A. Macrophages Are Polarized toward an Inflammatory Phenotype by their Aged Microenvironment in the Human Skin. J Invest Dermatol. 2022; 142:3136–45.e11. https://doi.org/10.1016/j.jid.2022.06.023 PMID:35850208
2. Wiśniewski JR. Filter-Aided Sample Preparation for Proteome Analysis. Methods Mol Biol. 2018; 1841:3–10. https://doi.org/10.1007/978-1-4939-8695-8_1 PMID:30259475
3. Skowronek P, Thielert M, Voytik E, Tanzer MC, Hansen FM, Willems S, Karayel O, Brunner AD, Meier F, Mann M. Rapid and In-Depth Coverage of the (Phospho-)Proteome With Deep Libraries and Optimal Window Design for dia-PASEF. Mol Cell Proteomics. 2022; 21:100279. https://doi.org/10.1016/j.mcpro.2022.100279 PMID:35944843
4. Demichev V, Messner CB, Vernardis SI, Lilley KS, Ralser M. DIA-NN: neural networks and interference correction enable deep proteome coverage in high throughput. Nat Methods. 2020; 17:41–4. https://doi.org/10.1038/s41592-019-0638-x PMID:31768060
5. Love MI, Huber W, Anders S. Moderated estimation of fold change and dispersion for RNA-seq data with DESeq2. Genome Biol. 2014; 15:550. https://doi.org/10.1186/s13059-014-0550-8 PMID:25516281
6. Wickham H, François R, Henry L, Müller K, Vaughan D. dplyr: a grammar of data manipulation. R package version 1.1. 2. Computer software. 2023.
7. Dowle M, Srinivasan A. data. table: Extension of 'data. frame'. R package version. 2019; 1.
8. Gu Z, Eils R, Schlesner M. Complex heatmaps reveal patterns and correlations in multidimensional genomic data. Bioinformatics. 2016; 32:2847–9. https://doi.org/10.1093/bioinformatics/btw313 PMID:27207943
9. Kolberg L, Raudvere U, Kuzmin I, Adler P, Vilo J, Peterson H. g:Profiler-interoperable web service for functional enrichment analysis and gene identifier mapping (2023 update). Nucleic Acids Res. 2023; 51:W207–12. https://doi.org/10.1093/nar/gkad347 PMID:37144459
10. Szklarczyk D, Kirsch R, Koutrouli M, Nastou K, Mehryary F, Hachilif R, Gable AL, Fang T, Doncheva NT, Pyysalo S, Bork P, Jensen LJ, von Mering C. The STRING database in 2023: protein-protein association networks and functional enrichment analyses for any sequenced genome of interest. Nucleic Acids Res. 2023; 51:D638–46. https://doi.org/10.1093/nar/gkac1000 PMID:36370105
