# Supplementary material for: Systemic factors in young human serum influence in vitro responses of human skin and bone marrow-derived blood cells in a microphysiological co-culture system
Source: Aging (Albany NY). 2025 Jul 25;17(7):1784–809. doi: 10.18632/aging.206288 (PMC12339024; doi:10.18632/aging.206288)
Supplement: Supplementary Figures [file aging-17-206288-s002.pdf]

SUPPLEMENTARY FIGURES

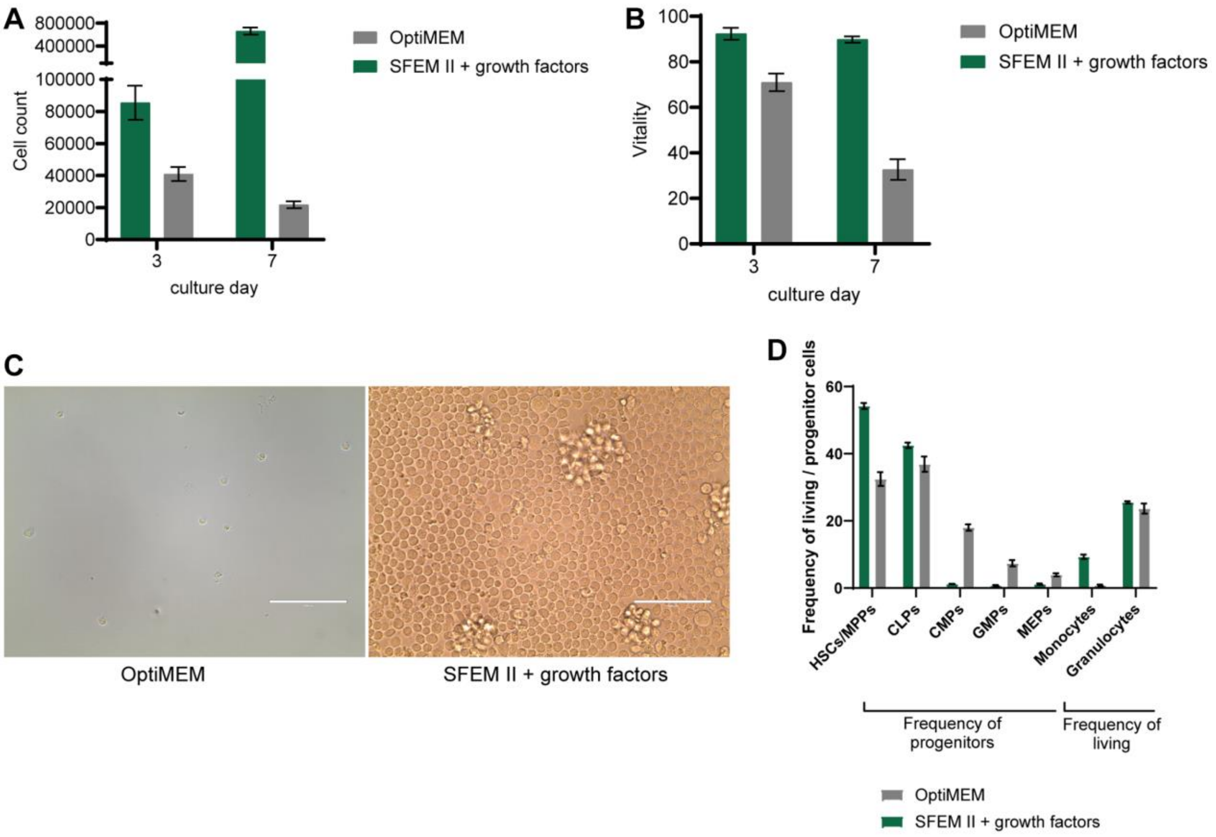

Supplementary Figure 1. CD34+ cells cultivation in OptiMEM vs. SFEM II. The CD34+ cells were either cultured in OpitMEM medium or SFEM II medium supplemented with 1 ng/mL GM-CSF, 1 ng/mL M-CSF, 50 ng/mL SCF, 100 ng/mL Flt3-L and 10 ng/mL TPO. (**A**) Cell count after 3 and 7 days of culture. (**B**) Vitality after 3 and 7 days of culture. (**C**) Cell morphology after 6 days of culture, scale bar 100 µm. (**D**) Cell differentiation after 7 days of culture, less than 1,000 cells measured for OptiMEM, results need to be handled with care. Data are shown as mean +/− SEM, *n* = 6.

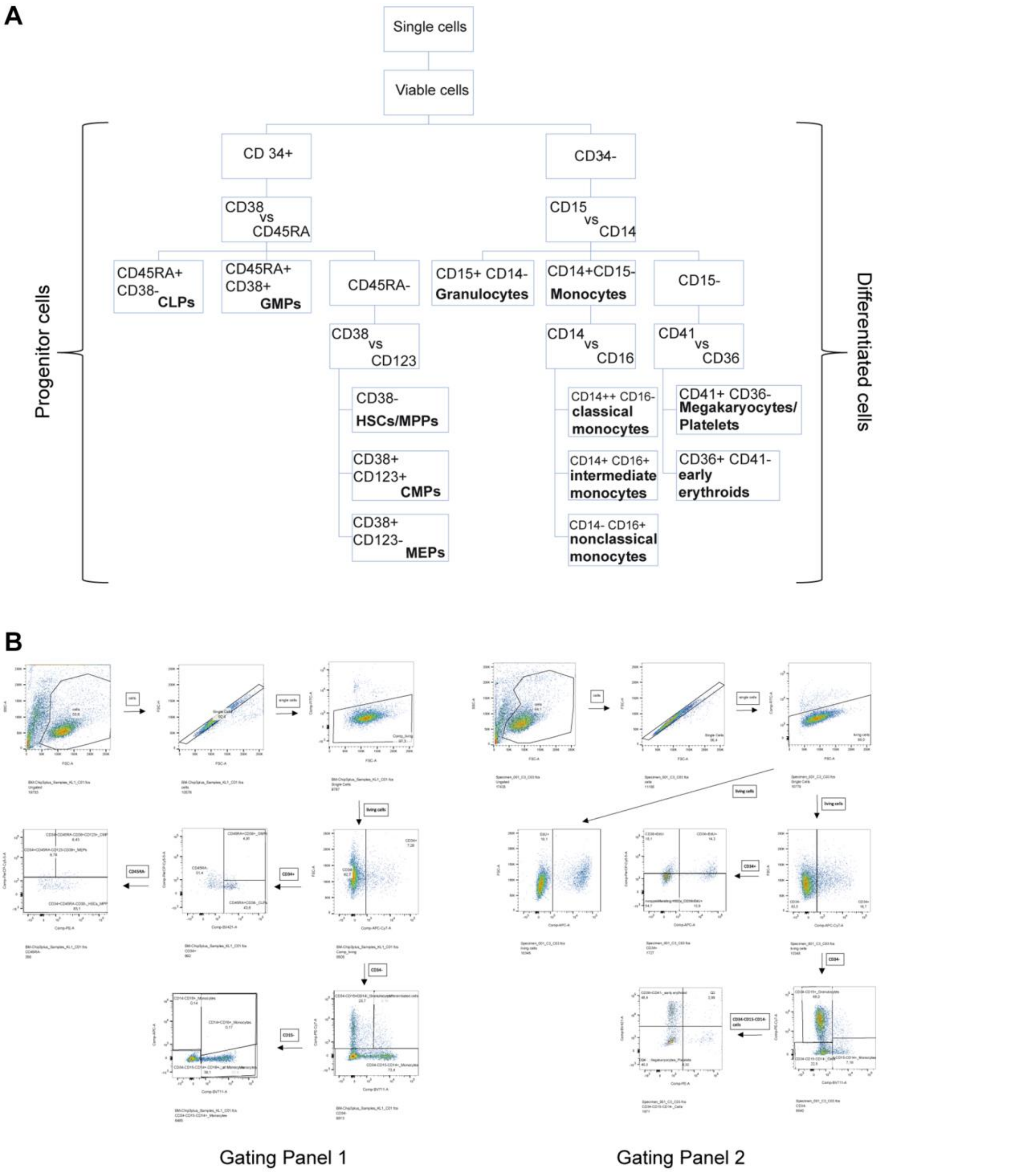

Supplementary Figure 2. Gating Strategy of flow cytometry analysis. (**A**) Gating tree showing the identification of different cell types based on their cell surface markers. CD34 was used to distinguish progenitor cells from more differentiated cells. CLPs, GMPs, HSCs/MPPs, CMPs and MEPs were identified within the progenitor cell population. On the other hand, granulocytes, megakaryocytes/platelets, early erythroid and granulocytes were examined. The monocytes were further classified into classical, intermediate and nonclassical monocytes. (**B**) To be able to distinguish that many cell populations, two different flow cytometry panels were used as shown.

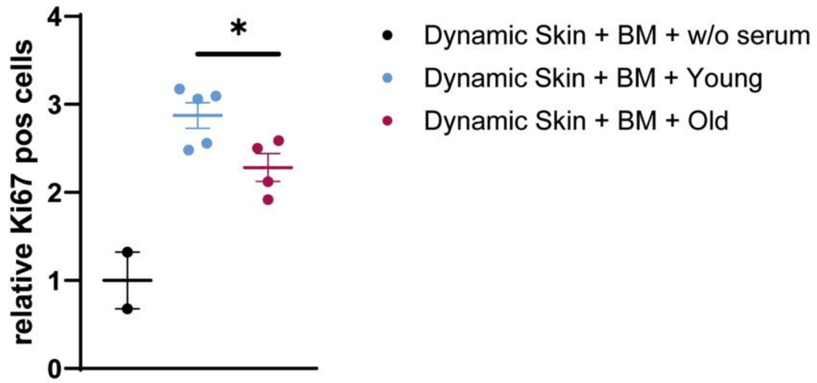

Supplementary Figure 3. Dynamic skin cultivation with human serum increases proliferation. Human skin models were dynamically co-cultured in the HUMIMIC Chip3 plus for three weeks either without serum, with young human serum or old human serum. The relative proportion of Ki67+ cells normalized to treatment without serum is depicted. Data are shown as mean +/− SEM, obtained from one experiment with *n* = 2–5.
