# Supplementary material for: Systemic factors in young human serum influence in vitro responses of human skin and bone marrow-derived blood cells in a microphysiological co-culture system
Source: Aging (Albany NY). 2025 Jul 25;17(7):1784–809. doi: 10.18632/aging.206288 (PMC12339024; doi:10.18632/aging.206288)
Supplement: Supplementary Tables 1 and 3-5 [file aging-17-206288-s003.pdf]

SUPPLEMENTARY TABLES

Supplementary Table 1. Age relevant gene panel of human skin dermis biopsies from the arm, study was performed with nine young (age 18 to 27) and nine old (age 61 to 72 years) participants, data published in "Characterization of transcriptome of human skin from different body areas und ages" by Elisabeth Wurzer.

Gene	Regulation old vs. young
DPP4	Up
DPT	Up
SOD3	Up
FBLN1	Up
DCN	Up
MMP2	Up
ELN	Up
TNFAIP6	Up
CTSK	Up
FBN1	Up
IGFBP6	Up
TIMP1	Up
BGN	Up
COL1A2	Up
PTGES	Up
VCAN	Up
VIM	Up
LOX	Up
EMILIN1	Up
IGDCC4	Up
HAS2	Up
TIMP2	Up
APOL1	Up
CD63	Up
THBS1	Up
SLPI	Up
LOXL1	Up
BAD	Up
CTSF	Up
TAGLN	Up
LMNA	Up
A2M	Up
LGALS3BP	Up
CDK4	Up
PTN	Up
ANAPC10	Up
ATG5	Up
TAF10	Up
IL1R1	Up
HES1	Up
ATM	Up

SMARCE1	Up
ARFIP2	Up
CDK16	Up
SIRT3	Up
CDK5RAP3	Up
SOD2	Up
APLP2	Up
PTGS2	Up
CERK	Up
GLO1	Up
UQCRB	Up
FAM32A	Down
HDGF	Down
ZFX	Down
LY6E	Down
NCOA6	Down
STAT3	Down
UBN1	Down
YIPF6	Down
NDUFV2	Down
AQP3	Down
HSPB1	Down
OCLN	Down
MRPL33	Down
EZH2	Down
KDM6B	Down
CTSD	Down
CEBPB	Down
UQCRC2	Down
VEGFA	Down
MTOR	Down
APOE	Down
CDKN1A	Down
PPARA	Down
ACSS2	Down

Supplementary Table 3. List of human serum, all purchased from Zen-Bio.

Category	Age	Lot	Cat. No
**Old**	62	SER101822K	HSER-CUSTOM_50 ml
	69	SER101822AB	HSER-CUSTOM_50 ml
	71	SER101822AJ	HSER-CUSTOM_50 ml
	61	SER101822AV	HSER-CUSTOM_50 ml
	69	SER101822AK	HSER-CUSTOM_50 ml
	66	SER101822AO	HSER-CUSTOM_50 ml
	59	SER101822BA	HSER-CUSTOM_50 ml
	60	SER101822S	HSER-CUSTOM_50 ml
	75	SER101822AQ	HSER-CUSTOM_50 ml

Young	69	SER101822Y	HSER-CUSTOM_50 ml
	21	SER101822BT	HSER-CUSTOM_50 ml
	24	SER101822BY	HSER-CUSTOM_50 ml
	25	SER101822BG	HSER-CUSTOM_50 ml
	26	SER101822CD	HSER-CUSTOM_50 ml
	26	SER101822BP	HSER-CUSTOM_50 ml
	27	SER101822BC	HSER-CUSTOM_50 ml
	29	SER101822G	HSER-CUSTOM_50 ml
	29	SER101822F	HSER-CUSTOM_50 ml
	29	SER101822BM	HSER-CUSTOM_50 ml
	30	SER101822BQ	HSER-CUSTOM_50 ml

Supplementary Table 4. List of antibodies used for flow cytometry, all purchased from BioLegends.

Antibody	Clone	Conjugate	Dilution	Cat. No
Anti-human CD14, IgG1, κ	63D3	Brilliant Violet 711™	1:20	367140
Anti-human CD15 (SSEA-1), IgM, κ	HI98	PE/Cyanine7	1:20	301924
Anti-human CD16, IgG1, κ	3G8	APC	1:20	302012
Anti-human CD34, IgG1, κ	581	APC/Cyanine7	1:20	343514
Anti-human CD38, IgG1, κ	HIT2	PerCP/Cyanine5.5	1:20	980314
Anti-human CD45RA, IgG2b, κ	HI100	Brilliant Violet 421™	1:20	304130
Anti-human CD123, IgG1, κ	6H6	PE	1:20	306006
Anti-human CD13, IgG1, κ	WM15	Brilliant Violet 711™	1:20	301722
Anti-human CD41, IgG1, κ	HIP8	PE/Cyanine7	1:20	303718
Anti-human CD71, IgG1, κ	CY1G4	PerCP/Cyanine5.5	1:20	334114
Anti-human CD36, IgG1, κ	5-271	Brilliant Violet 421™	1:20	336230
Anti-human CD235a, IgG1, κ	HI267	APC	1:20	349114
Anti-human CD229, IgG1, κ	Hly-9.1.25	PE	1:20	326108

Supplementary Table 5. List of antibodies used for immunofluorescence staining

Antibody	Host	Dilution	Manufacturer	Cat. No
Anti-Ki67	Rabbit	1:300	Abcam	ab15580
Collagen IV	Mouse	1:100	Invitrogen	14-9871-82
Anti-Cytokeratin 14	Mouse	1:100	Abcam	ab7800
Keratin 10	Mouse	1:100	Antikoerper-onlin.de	ABIN1301947
