# Supplementary material for: Systemic factors in young human serum influence in vitro responses of human skin and bone marrow-derived blood cells in a microphysiological co-culture system
Source: Aging (Albany NY). 2025 Jul 25;17(7):1784–809. doi: 10.18632/aging.206288 (PMC12339024; doi:10.18632/aging.206288)
Supplement: Supplementary Table 2 [file aging-17-206288-s004.docx]

**Supplementary Tables 2. Primary Data of Figure 5.**

**Supplementary Table 2.1**. % of Ki67 positive cells, Ki67 staining fibroblasts

|  | **D1 - 73y** | **D2 - 77y** | **D3 - 68y** | **D4 - 64y** | **D5 - 63y** | **D6 - 66y** | **D7 - 69y** | **D8 - 63y** | **D9 - 67y** | **D10 - 64y** | **D11 - 77y** | **D12 - 68y** | **D13 - 78y** | **D14 - 64y** | **D15 - 67y** |
| --- | --- | --- | --- | --- | --- | --- | --- | --- | --- | --- | --- | --- | --- | --- | --- |
| **Control** | 14,66 | 19,42 | 37,12 | 32,96 | 25,15 | 25,33 | 3,79 | 5,38 | 5,01 | 15,87 | 7,21 | 17,30 | 18,90 | 30,88 | 17,00 |
| **CST7** | 57,92 | 21,22 | 61,48 | 51,81 | 36,83 | 34,11 | 29,17 | 39,64 | 40,01 |  | 9,41 |  | 21,39 |  | 22,56 |
| **IL1RN** | 27,97 | 29,06 | 60,78 | 28,63 | 33,89 | 41,60 | 18,37 | 26,00 | 15,13 | 21,42 | 11,32 |  |  |  | 19,06 |
| **CD55** | 53,59 | 26,63 | 54,01 | 49,08 | 26,64 | 45,85 |  |  |  | 38,90 | 28,92 | 45,45 | 40,98 | 44,96 | 53,17 |
| **SPINT1** | 35,28 | 22,17 | 58,62 | 38,47 | 40,02 | 48,01 | 33,87 | 41,93 | 44,14 | 29,35 | 17,45 |  | 25,54 |  | 37,74 |
| **MMP9** | 29,20 | 35,13 | 56,24 | 77,60 | 87,42 | 46,23 | 13,64 | 33,14 | 26,82 | 35,62 | 31,46 |  |  |  | 20,31 |
| **FCAR** | 34,26 | 41,39 | 60,92 | 52,73 | 61,97 | 58,20 | 14,56 | 37,84 | 17,18 |  |  |  |  |  |  |
| **CHI3L** | 43,32 | 41,31 |  |  |  |  |  |  |  | 23,04 | 12,21 |  |  |  | 17,39 |
| **GDF-11** | 14,64 | 45,85 |  |  |  |  | 28,73 |  |  | 20,03 | 12,37 |  | 21,32 | 45,70 | 25,32 |

**Supplementary Table 2.2**. % of Ki67 positive cells, Ki67 staining keratinocytes

|  | **D1 - 73y** | **D2 - 70y** | **D3 - 73y** | **D4 - 73y** | **D5 - 67y** | **D6 - 76y** | **D7 - 72y** | **D8 - 62y** | **D9 - 65y** | **D10 - 67y** | **D11 - 66y** | **D12 - 76y** | **D13 - 69y** | **D14 -65y** | **D15 - 59y** | **D16 - 67y** | **D17 - 68y** | **D18 - 63y** | **D19 - 72y** | **D20 - 72y** |
| --- | --- | --- | --- | --- | --- | --- | --- | --- | --- | --- | --- | --- | --- | --- | --- | --- | --- | --- | --- | --- |
| **Control** | 62,44 | 90,13 | 80,98 | 61,27 | 74,17 | 39,68 | 14,82 | 37,16 | 34,78 | 31,17 | 38,68 | 62,83 | 79,82 | 77,95 | 44,88 | 37,44 | 22,41 | 17,47 | 13,26 | 5,80 |
| **CST7** | 74,74 | 87,23 | 64,61 | 58,07 | 78,13 | 43,54 | 37,93 | 31,30 | 35,60 | 29,42 | 59,83 | 67,66 | 86,65 | 84,68 | 37,99 | 41,92 | 28,84 | 27,11 | 19,79 | 13,16 |
| **IL1RN** | 67,07 | 66,40 | 69,69 | 64,44 | 77,27 | 59,09 | 30,56 | 30,37 | 44,84 | 40,66 |  | 64,78 | 87,68 | 82,65 | 38,15 | 29,95 | 20,39 | 15,20 | 19,37 | 10,64 |
| **CD55** | 70,13 | 79,94 | 79,70 | 53,74 | 72,15 | 35,58 | 24,37 | 25,50 | 36,28 | 22,76 |  |  |  |  | 54,79 | 46,74 | 31,03 | 10,83 | 17,05 | 8,76 |
| **SPINT1** | 79,41 | 74,09 | 69,08 | 61,59 | 63,22 | 36,55 | 23,64 | 31,36 | 30,72 | 32,67 |  | 69,29 | 85,66 | 83,08 | 50,77 | 36,61 | 9,50 | 21,13 | 20,52 | 17,72 |
| **MMP9** | 76,35 | 67,48 | 60,37 | 55,16 | 65,03 | 25,85 | 28,95 | 31,83 | 40,72 | 35,59 | 39,10 |  |  |  | 38,60 | 35,94 | 13,89 | 17,82 | 14,79 | 5,36 |
| **FCAR** | 67,74 | 71,62 | 76,80 | 60,67 | 66,16 | 26,99 | 30,97 | 26,16 | 32,02 | 45,27 |  | 61,79 | 81,71 | 85,68 |  |  |  |  |  |  |
| **CHI3L** | 71,36 | 81,67 | 70,03 | 55,23 |  |  |  | 29,67 | 41,50 | 40,73 |  |  |  |  | 33,66 | 33,70 | 15,24 | 19,92 | 23,68 | 8,18 |
| **GDF-11** | 69,13 | 95,52 | 83,74 | 59,82 |  |  |  | 55,77 | 57,95 | 64,32 |  | 65,54 | 85,01 | 80,00 | 39,49 | 48,67 | 28,46 | 24,28 | 17,91 | 6,26 |

**Supplementary Table 2.3**. hyaluronic acid [ng/mL], in supernatant of fibroblasts

|  | **D1 - 64y** | **D2 - 63y** | **D3 - 66y** | **D4 - 68y** | **D5 - 78y** | **D6 - 64y** | **D7 - 67y** |
| --- | --- | --- | --- | --- | --- | --- | --- |
| **Control** | 47,85 | 31,65 | 69,03 | 102,23 | 24,75 | 66,63 | 37,75 |
| **CST7** | 60,47 | 45,24 | 49,50 | 110,08 | 26,94 | 74,71 | 59,44 |
| **IL1RN** | 54,38 | 25,28 | 82,63 | 117,71 | 57,71 | 90,28 | 36,39 |
| **CD55** | 74,95 | 51,80 | 111,16 |  | 126,46 | 114,00 | 160,60 |
| **SPINT1** | 72,31 | 56,33 | 65,26 | 94,67 | 39,07 | 82,47 | 20,42 |
| **MMP9** | 104,50 | 67,48 | 70,17 | 291,77 | 58,50 | 143,04 | 63,24 |
| **FCAR** | 51,73 | 38,62 | 84,42 |  |  |  |  |
| **CHI3L** | 73,92 | 52,90 | 89,91 | 132,44 | 38,71 | 62,64 | 60,22 |
| **GDF-11** | 99,94 | 47,84 | 161,34 | 110,24 | 57,76 | 39,21 | 44,99 |

**Supplementary Table 2.4**. procollagen 1 [ng/mL], in supernatant of fibroblasts

|  | **D1 - 64y** | **D2 - 63y** | **D3 - 66y** | **D4 - 68y** | **D5 - 78y** | **D6 - 64y** | **D7 - 67y** |
| --- | --- | --- | --- | --- | --- | --- | --- |
| **Control** | 340,46 | 337,45 | 348,48 | 474,67 | 514,62 | 439,14 | 484,81 |
| **CST7** | 519,78 | 710,64 | 645,27 |  | 550,34 | 466,44 | 538,14 |
| **IL1RN** | 479,46 | 201,62 | 360,90 | 526,03 | 510,86 | 459,52 | 415,08 |
| **CD55** | 888,25 | 925,53 | 852,57 |  |  |  |  |
| **SPINT1** | 864,88 | 609,82 | 534,04 |  | 523,81 | 438,84 | 542,15 |
| **MMP9** | 529,06 | 702,15 | 575,69 | 432,06 | 493,81 |  | 434,28 |
| **FCAR** | 438,59 | 338,16 | 387,93 |  |  |  |  |
| **CHI3L** | 819,38 | 830,55 | 1102,42 | 480,91 | 523,07 | 451,79 | 486,41 |
| **GDF-11** | 1064,90 | 609,82 | 758,69 | 527,65 | 573,94 | 560,26 | 523,86 |

**Supplementary Table 2.5**. % of fibroblasts differentiated into adipocytes

|  | **D1 - 73y** | **D2 - 73y** | **D3 - 64y** | **D4 - 63y** | **D5 - 66y** | **D6 - 69y** | **D7 - 63y** | **D8 - 67y** | **D9 - 76y** | **D10 - 77y** | **D11 - 68y** | **D12 - 78y** | **D13 - 64y** | **D14 - 67y** |
| --- | --- | --- | --- | --- | --- | --- | --- | --- | --- | --- | --- | --- | --- | --- |
| **Control** | 19,12 | 28,48 | 4,08 | 7,17 | 1,54 | 7,17 | 2,92 | 0,41 | 2,12 | 33,30 | 34,12 | 49,98 | 45,79 | 35,52 |
| **CST7** | 22,72 | 23,10 | 6,81 | 7,95 | 1,35 | 9,42 | 4,07 | 0,92 | 3,15 | 40,19 |  |  | 46,55 | 35,59 |
| **IL1RN** | 33,47 | 44,04 | 6,83 | 10,29 | 1,11 | 8,93 | 4,06 | 1,13 | 3,09 | 44,21 | 33,10 | 53,98 | 62,73 | 43,34 |
| **CD55** | 23,19 | 55,36 | 7,89 | 12,02 | 1,54 |  |  |  | 2,16 | 41,00 |  | 60,48 | 51,29 | 46,18 |
| **SPINT1** | 23,62 | 37,45 | 5,74 | 7,23 | 1,23 | 8,04 | 3,13 | 1,01 | 5,29 | 46,50 | 45,12 | 49,84 | 56,15 | 40,31 |
| **MMP9** | 19,25 | 57,18 | 5,16 | 6,62 | 1,44 | 10,11 | 2,54 | 0,35 | 3,73 | 55,51 | 35,83 | 55,98 | 84,71 | 47,60 |
| **FCAR** | 34,04 | 60,70 | 7,33 | 9,01 | 1,91 | 9,57 | 4,06 | 0,67 | 1,80 | 45,40 |  |  |  |  |
| **CHI3L** | 30,04 | 69,01 | 7,41 | 10,35 | 1,90 |  |  |  | 5,28 | 42,89 | 33,78 | 45,48 | 66,86 | 46,89 |
| **GDF-11** |  | 42,85 | 5,59 | 3,85 | 0,37 | 3,87 | 2,60 | 0,33 |  | 19,46 | 20,82 | 10,00 | 26,56 | 6,04 |

**Supplementary Table 2.6**. ratio green (525nm)/red (590 nm), mitochondrial membrane potential of fibroblasts

|  | **D1 - 73y** | **D2 - 77y** | **D3 - 68y** | **D4 - 64y** | **D5 - 63y** | **D6 - 66y** | **D7 - 68y** | **D8 - 78y** | **D9 - 64y** | **D10 - 67y** |
| --- | --- | --- | --- | --- | --- | --- | --- | --- | --- | --- |
| **Control** | 0,74 | 0,68 | 0,73 | 0,83 | 1,26 | 1,26 | 0,611 | 0,644 | 0,665 | 0,645 |
| **CST7** | 0,78 | 0,72 | 0,74 | 0,83 | 1,18 | 1,54 | 0,714 | 0,724 | 0,598 | 0,986 |
| **IL1RN** | 0,73 | 0,66 | 0,71 | 1,08 | 1,16 | 1,45 | 0,729 | 0,793 | 0,842 | 0,695 |
| **CD55** |  |  |  | 0,84 | 1,17 | 1,35 | 0,612 | 0,613 | 0,616 | 0,579 |
| **SPINT1** | 0,73 | 0,67 | 0,69 | 0,83 | 1,06 | 1,44 | 0,682 | 0,638 | 0,575 | 0,594 |
| **MMP9** | 0,70 | 0,69 |  | 0,94 | 1,49 | 1,55 | 0,700 | 0,788 | 0,915 | 0,675 |
| **FCAR** | 0,87 | 0,69 | 0,72 | 0,81 | 1,29 | 1,24 |  |  |  |  |
| **CHI3L** |  |  |  | 0,82 | 1,30 | 1,52 | 0,730 | 0,776 | 0,826 | 0,702 |
| **GDF-11** | 0,76 | 0,77 |  | 1,32 | 1,26 | 1,38 | 0,649 | 0,735 | 0,719 | 0,705 |
